# Improvement of ovarian function in a premature ovarian failure mouse model using *Vitex agnus-castus* extract

**DOI:** 10.5935/1518-0557.20240101

**Published:** 2025

**Authors:** Zeinab Soleimany, Fatemeh Siadat, Mona Farhadi, Zeinab Sadat Mirshaby, Zahra Sanadgol, Hossein Eyni

**Affiliations:** 1 Department of Biology, North Tehran Branch, Islamic Azad University, Tehran, Iran; 2 Department of Microbiology, Karaj Branch, Islamic Azad University, Karaj, Iran; 3 Department of Biochemistry, Islamic Azad University, Shahr Ghods, Tehran, Iran; 4 Stem Cell and Regenerative Medicine Research Center, Department of Anatomy, School of Medicine, Iran University of Medical Sciences, Tehran, Iran

**Keywords:** POF, premature ovarian failure, Vitex agnus castus, follicle

## Abstract

**Objective:**

Premature ovarian failure (POF) leads to infertility. Numerous researchers have endeavored to enhance ovarian function through antioxidant interventions. Extract from *Vitex agnus-castus* (VAC) has demonstrated a protective effect. Therefore, the objective of this study was to investigate the amelioration of ovarian function following VAC treatment in a POF mouse model.

**Methods:**

In this investigation, 30 female NMRI mice were categorized into control, POF model (cyclophosphamide 120 mg/kg I.P), and experimental groups (100, 300, and 500 of VAC extract). Parameters such as body weight, vaginal smears, and follicular evaluation were examined. FSH, estradiol levels, free radicals, and the expression of the FMR1 gene were assessed.

**Results:**

The microscopic assessment revealed that POF induced morphological alterations in ovarian tissue, whereas VAC treatment significantly ameliorated ovarian tissue conditions. The follicles number exhibited a significant reduction in the POF group; however, VAC led to an increase in follicular count and elevated estradiol levels in the treatment groups. Serum FSH levels displayed an elevation in the POF group, whereas the treatment groups exhibited a substantial reduction in FSH levels compared to the POF group. The expression of the FMR1 gene demonstrated upregulation in the POF group compared to the control group (*p*<0.05). Moreover, this expression significantly decreased in the 500-dose VAC group compared to the POF group (*p*<0.001). ROS generation exhibited a significant increase in the POF group, which was conversely mitigated in all experimental groups..

**Conclusions:**

Our findings underscore the potential of this extract to ameliorate POF symptoms, however, further investigations are needed.

## INTRODUCTION

The number of follicles in a female infant is fixed at birth, and the available reserve is the only source of fertilized eggs during the reproductive period. In normal ovarian folliculogenesis, unresponsive follicles may atrophy due to apoptosis. Accelerated atresia may cause an insufficient supply of follicles for ovulation, resulting in premature ovarian failure and infertility. Premature ovarian failure (POF) is characterized by the follicular count before the age of 40, affecting 1-2% of women of reproductive age and influenced by ethnicity (Mashayekhi *et al*., 2021). The prevalence of POF increases with age, occurring in 1 out of 10,000 women in their 20s, 1 out of 1,000 women in their 30s, and 1 out of 100 women in their 40s (Graff & Christin-Maitre, 2019). Possible causes of POF include genetic and autoimmune disorders, environmental factors, pathogenic and idiopathic conditions (Mashayekhi *et al*., 2021), with the idiopathic POF having an unknown pathologic cause in most cases (Maclaran & Panay, 2011). POF usually presents as amenorrhea and infertility. The main symptom is the absence of regular menstrual cycles, and the diagnosis is confirmed by an increase in follicle-stimulating hormone and a decrease in the level of anti-Müllerian hormone (AMH), which indicates primary ovarian failure (Luisi *et al*., 2015). Also, the low level of estrogen is one of the other clinical symptoms of this condition (Woad *et al*., 2006). Anticancer drugs, such as cyclophosphamide and Busulfan, have been shown to be very harmful to ovarian follicles. Therefore, fertility and ovarian function preservation should be the main consideration of chemotherapy in women of reproductive age (Lv *et al*., 2021). Chemotherapy and radiotherapy used to treat malignant diseases are the most commonly known causes of POF, reducing the number and affecting the structure and function of oocytes and granulosa cells (Nippita & Baber, 2007).

POF occurs through two mechanisms: follicular dysfunction and follicle depletion. Follicular dysfunction occurs when a pathological process prevents the normal function of the follicles. Follicle depletion occurs when there is no primordial follicle left in the ovary due to the failure to develop an adequate initial pool of primordial follicles in utero, rapid consumption of follicles, or autoimmune destruction. Some but not all patients with POF experience estrogen deficiency symptoms, including vasomotor symptoms, sleep disturbance, and dyspareunia related to vaginal dryness (Nelson, 2009). However, until now, there is no cure for POF. Women suffering from POF are severely affected physically and mentally and have to face infertility, amenorrhea, osteoporosis, some cardiovascular diseases, etc. POF is mostly linked to decreased antral follicle and granulosa cell activity (He *et al*., 2018). The Verbenaceae family tree known by the popular name VAC is the chaste tree, which grows throughout Central Asia, the Mediterranean, and southern Europe. It is also harvested in various locations (Niroumand *et al*., 2018). VAC contains bioactive compounds, including essential fatty acids, volatile oil, alkaloid, progestin, flavonoids, iridoid glycosides, and phytosteroids, with various biological properties, including anti-tumor, antioxidant, and anti-inflammatory properties. Using of VAC in medicine has a long history. It is used to control libido, uterine diseases, and wound healing. In addition, the compounds of this plant soothe symptoms such as headaches, syphilis, flu, and gastric diarrhea. It also has a large number of biological properties, including anti-tumor properties, antioxidant and anti-inflammatory properties (Alamoudi & Bakrshoom, 2021).

Research into potential treatments for POF is ongoing, with a recent study investigating the effects of *Vitex agnus castus* (VAC) extract in a mouse model of POF. VAC is a herbal remedy that has traditionally been used to regulate menstrual cycles and improve fertility. The study found that VAC extract was able to improve the number and quality of follicles in the ovaries of POF mice, suggesting that it could have potential as a treatment for POF in humans. This article will explore the causes and symptoms of POF, the role of follicles in ovarian function, and the potential benefits of VAC extract for treating POF in more detail, drawing on recent research and expert opinions.

Evidence suggests that VAC extract is attached to dopamine receptors (D2) in the hypothalamus and anterior pituitary, inhibiting prolactin secretion, it appears to affect other endocrine glands, including increased progesterone secretion and induction of natural formation of the body and may increase female fertility. VAC fruits are used to treat women’s problems, including menstrual disorders, premenstrual symptoms, menopausal symptoms, breastfeeding disorder, acne, bodies, and infertility (Dugoua *et al*., 2008).

The Fragile-X-Mental-Retardation-1 (FMR1) gene contains a CGG repetition in the non-translated area of exon 1 and was the first disease gene shown to contain a duplicate element that can extend to more than ten times the normal size, resulting in a clinical phenotype (Murray, 2000). The CGG nucleotides naturally consist of 5-44 reps, with 45-54 repetitions of the medium allele allowing translation and expression of the FMR1 gene. When the CGG repetition expands between 55 and 200 (pre-maturation -PM), the gene continues to transcribe more mRNA. Many studies suggest that the FMR1 gene is about 6 % of POF cases (Nagarathinam *et al*., 2021). The FMR1 gene is involved in three different syndromes: fragile X syndrome, POF, and Fragile X-associated tremor/ataxia syndrome (FXTAS) at an older age (Oostra & Willemsen, 2003). The FMR1 gene is a key gene for ovarian storage and folliculogenesis (Rehnitz *et al*., 2018). There is currently no cure for POF, and women with POF face infertility, amenorrhea, osteoporosis, and cardiovascular diseases (He *et al*., 2018).

This study aimed to investigate the effects of *Vitex agnus-castus* extract on cyclophosphamide-induced premature ovarian failure (POF) in female NMRI mice. The study specifically aimed to evaluate the serum level of sex hormones, histopathological changes in the ovaries, oxidative stress markers, and FMR1 gene expression in the experimental groups compared to the control and POF model groups.

## MATERIALS AND METHODS

### Extract preparation

Involved buying 250 gr of the *Vitex agnus-castus* (Giyahkala, Iran), powdering it, and then mixing it with 200 cc of 96% ethanol hydroalcoholic solvent. To separate the alcohol in the extract, the resultant solution was put inside the rotary evaporator after 48 hours.

### Study groups

In this study, 6 to 8-week-old adult female NMRI mice were used. All animal experiments performed, comply with the Animal Research Reporting of In Vivo Experiments (ARRIVE) guidelines and are carried out by the U.K. Animals and associated guidelines. They were kept in the animal house of Iran University of Medical Science under controlled temperature (20 to 25°C) and a 12/12hour dark/light cycle. The mice were divided into three groups (N=6): a control group without any treatment, a POF model group that induced by injection of 120 mg/ kg of cyclophosphamide (Irandarouk, Iran) (Wang *et al*., 2022) for 14 days, based on the specified chemotherapy protocol, and experimental groups. Experimental group 1 was administered gavage with a combination of cyclophosphamide and 100 mg/kg of VAC, experimental group 2 was given cyclophosphamide and 300 mg/kg of VAC, and experimental group 3 was given a combination of cyclophosphamide and 500 mg/kg VAC. The *Vitex agnus-castus* extract was administered to the mice for 21 days (Berrani *et al*., 2021). All mice were anesthetized, and blood samples were taken from their heart to assay the serum levels of sex hormones. The ovaries were then removed, and tissue sections were prepared and stained with H&E. Another group of ovaries was stored at -70°C for the evaluation of reactive oxidative stress (ROS) and FMR1 gene expression analysis.

### Vaginal smears collecting sample

Vaginal smears were collected from all groups. To reduce stress, mice were kept in their environment for 4 days after the end of the treatment period. The smear was collected a few days before the injection of cyclophosphamide. 50 microliters of normal saline (at room temperature) were injected into the vagina, and a few slides of liquid were prepared for microscopic evaluation. The estrous cycle was determined based on the presence or absence of leukocytes, keratinized epithelial cells, and nucleated epithelial cells. Changes in the composition and population of cells can indicate fundamental changes in the events of endocrine secretions of reproductive hormones and changes in the hypothalamic-pituitary axis of the ovary.

### Tissue preparation

Following the experimental treatment, an IP dose of ketamine 100 mg/kg and xylazine 10 mg/kg was used to anaesthetise each mouse, and then humanely killed. Their ovarian tissues were surgically removed and fixed in a 10% formalin buffer solution for one week.

### H&E staining

The fixed tissues were then dehydrated and embedded in paraffin wax. Thin sections of 5 microns were cut and mounted on glass slides, which were subsequently stained with hematoxylin and eosin (Merck). The stained slides were examined under a light microscope to stage the follicles based on their morphological characteristics, including primordial, primary, secondary, and atretic follicles.

### Hormone assay preparation

Hormone assay preparation involved anesthetizing the animals and collecting blood samples from the heart using a 2CC or 5CC syringe. The blood was then collected in microtubes and allowed to clot for a short period. After centrifugation, the supernatant was separated and stored in a refrigerator at -20°C until further analysis. Estradiol and Follicle-stimulating hormone (FSH) hormone levels were measured using the NAVAND LAB KIT.

### ROS assay preparation

To prepare the ROS assay, the tissue sample was washed with cold PBS and then 500-1000 µl of lysing buffer from the Navand Lab Kit (Iran) was added. The tissue was lysed using a homogenizer and centrifuged at 10,000 rpm for 10 minutes. The resulting supernatant was collected and used as a sample. For the assay, 30 µl of either the standard or the sample was added to a well, followed by the addition of 200 µl of Reagent 1, which was then shaken. Subsequently, 10 µl of Reagent 2 was added to the sample, and the mixture was incubated for 20 minutes. After the 20-minute incubation period, the optical absorption of the sample was read at a wavelength of 490 nm.

### Gene expression preparation

Total RNA was extracted from snap-frozen ovaries using the RNX-Plus Kit (Sinaclon, Iran) according to the manufacturer’s protocol. The extracted RNA concentration was measured using a Nano-drop spectrophotometer to confirm successful extraction. The cDNA was synthesized using a Thermofisher k1621 kit from America.

### Statistical analysis

**Figure 1:**
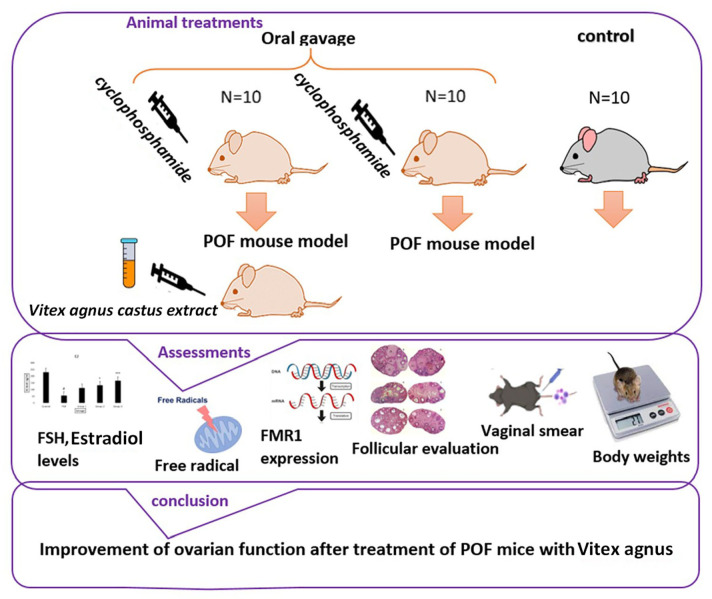
Schematic diagram of study.

Data were expressed as mean ± standard deviation. The Statistical Package for the Social Sciences (SPSS) software (v. 18, SPSS Inc., USA) was used to compare data via Analysis of Variance (ANOVA) and independent sample t-tests. *P* values <0.05 were deemed statistically significant (Figure 1).

## RESULTS

### Estrous cycle study

**Figure 2:**
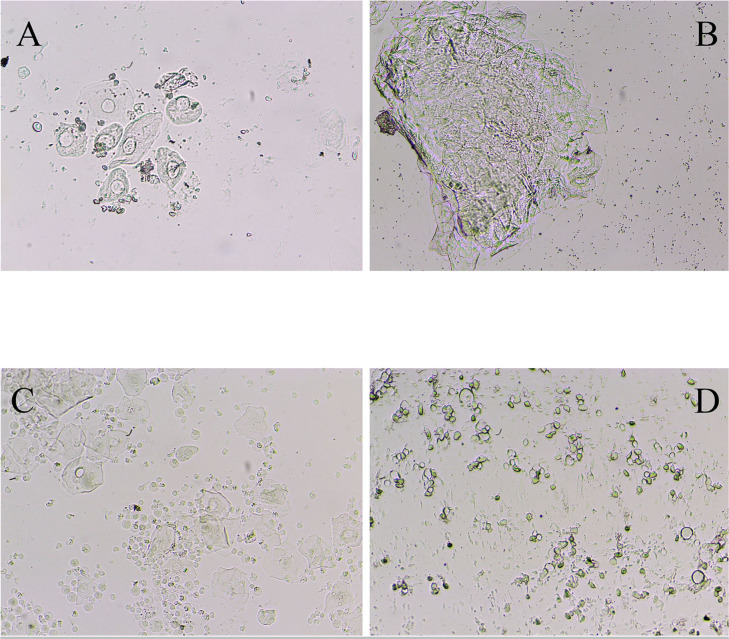
Vaginal smear sample of the control group (A=Prostrust, B=Strus, C=Metstrus, D=Distrus).

The study aimed to examine the impact of chemotherapy drug injection on the estrous cycle of control and POF mice. The results showed that the control group exhibited all four stages of the estrous cycle regularly (Figure 2).

**Figure 3:**
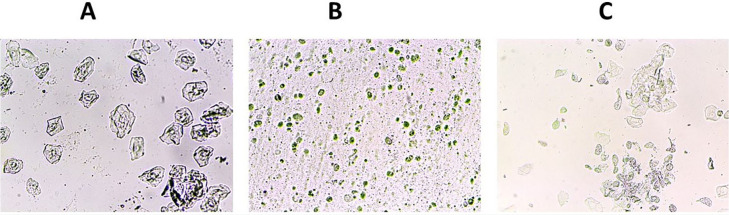
Vaginal smear sample of mice after 12 days of IP injection of cyclophosphamide (A=Prostrust, C=Metstrus, D=Distrus) Estrous stage was not observed in the patient group.

However, the POF group showed an irregular and prolonged estrous cycle compared to the control group. Furthermore, the ovulation phase was not observed in the POF group, which is essential for female mice to conceive (Figure 3). These findings suggest that chemotherapy treatment may have adverse effects on the reproductive health of female mice, leading to irregular and prolonged estrous cycles and the inhibition of ovulation.

### Morphological evaluation of ovarian tissue

**Figure 4:**
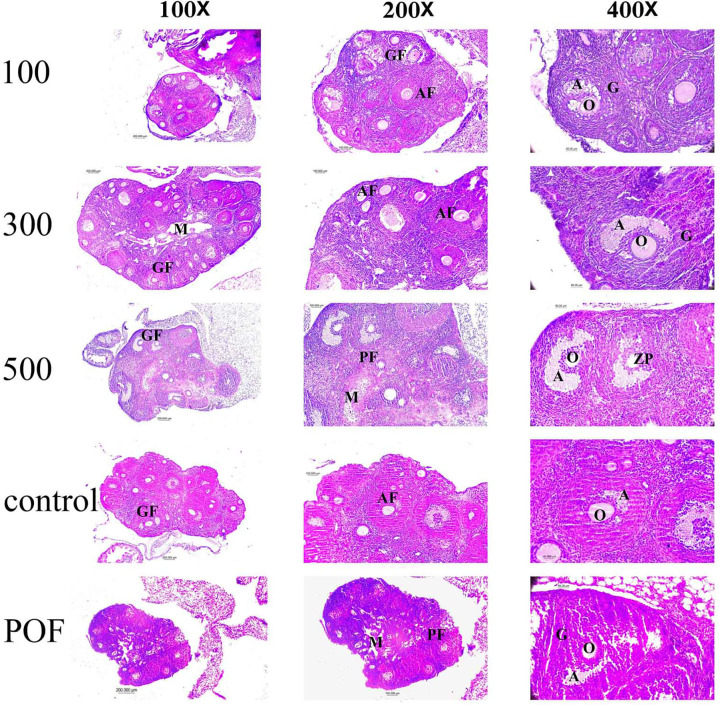
Morphological Evaluation of Ovarian Tissue Using hematoxylin-eosin staining, Group 100:POF+ Gavage 100 Dose of *Vitex agnus-castus* extract for 20 days, Group 300:POF+ Gavaj 300 Dose of Vitex agnus-castus extract for 20 days, Group 500: POF+500 Dosage Gauge 500 Extract *Vitex agnus-castus* extract for 20 days, control Group (no treatment was done), POF Group: Injectable injecting of 100 doses of cyclophosphamide for 14 days.

**Figure 5:**
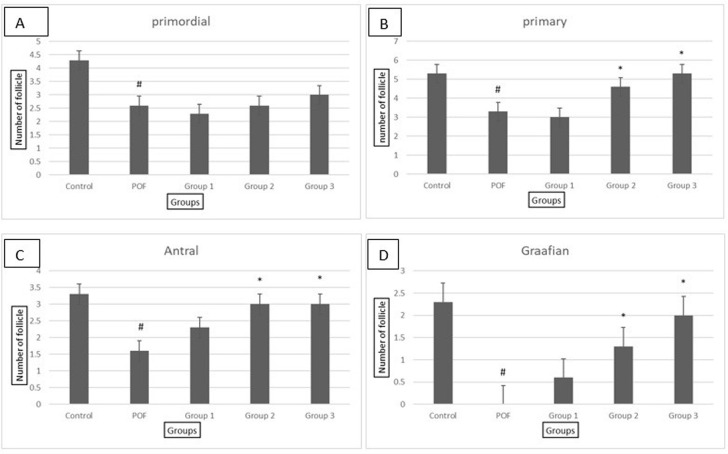
Evaluation of the number of ovarian follicles in all groups, group 1: POF+ 100 dose *of Vitex agnuscastus*, group 2: POF+ 300 dose of *Vitex agnus-castus*،, Group 3: POF+ 500 dose of Vitex agnus-castus, #: significant with the control group, *: significant with the patient group A-number of the primordial follicle in all groups, B-number of the primary follicle in all groups, C-number of Antral follicle in all groups and D- number of Graafian follicle in all groups.

The analysis of ovarian tissue morphology revealed significant differences between the POF and control groups. The number of follicles, particularly antral and Graafian follicles, was significantly lower in the ovaries of the POF group compared to the control group (Figures 4, 5A). However, in the experimental groups that received doses of 100, 300, and 500 mg/kg of the *Vitex agnuscastus* (VAC) extract, the number of follicles increased in a dose-dependent manner, with a significant increase observed in the doses of 300 and 500 mg/kg (Figure 5A). Further analysis showed that the number of primordial and primary follicles in the POF group was significantly lower than that in the control group (Figure 5B). However, in the experimental groups that received doses of 300 and 500 mg/kg of the extract, the number of primary follicles increased significantly compared to the POF group (Figure 4B). Moreover, the number of antral follicles and graft follicles in the POF group was significantly reduced compared to the control group (Figures 5C, 5D). However, treatment with the VAC extract at doses of 300 and 500 mg/kg resulted in a significant increase in the number of antral and graft follicles compared to the POF group (Figures 5C, 5D).

### Evaluation of Estradiol and FSH hormones

**Figure 6:**
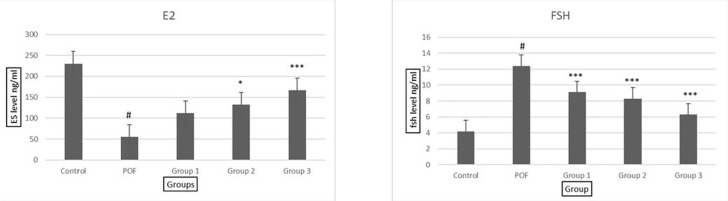
Examination of the serum levels of FSH and estradiol hormones in all groups, group 1: POF+100 dose of *Vitex agnus-castus*, group 2: POF+ 300 dose of *Vitex agnus-castus*, group 3: POF+ 500 dose of Vitex agnus-castus. A- The amount of estradiol hormone in all groups and B-The amount of FSH hormone in all groups.

The analysis of hormone levels revealed significant differences between the patient group and the healthy group. The amount of estradiol hormone was significantly lower in the patient group compared to the healthy group. However, in all treatment groups receiving the VAC extract, the amount of estradiol hormone increased compared to the patient group, with a more significant increase observed with higher doses of the extract. Notably, at a dose of 500 mg/kg, the amount of estradiol hormone approached that of the control group (Figure 6A).

On the other hand, the amount of FSH hormone was significantly higher in the patient group compared to the healthy group. However, in all treatment groups receiving VAC extract, the amount of FSH hormone decreased compared to the patient group. Furthermore, an increase in the dose of the plant extract resulted in a further decrease in the amount of FSH hormone. Remarkably, at a dose of 500 mg/kg, the amount of FSH hormone approached that of the control group (Figure 6B).

These findings suggest that the *Vitex agnus-castus* (VAC) may have a potentially positive effect on hormonal levels, specifically increasing estradiol and decreasing FSH levels, in the POF mice model. Further research is warranted to elucidate the underlying mechanisms of these hormonal changes and their implications for ovarian function.

### ROS generation

**Figure 7:**
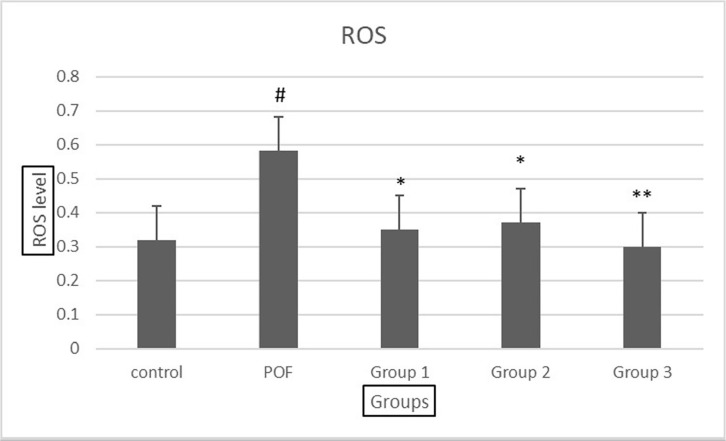
Comparison of the average level of oxidative stress in groups. group 1: POF+ 100 dose of Vitex agnus-castus, group 2: POF+ 300 dose of *Vitex agnus-castus*, group 3: POF+ 500 dose of *Vitex agnuscastus*.

The level of oxidative stress was significantly higher in the POF group compared to the control group, indicating increased ROS generation in the ovaries of POF mice. However, in the experimental groups receiving the VAC extract, the amount of oxidative stress decreased, with a more significant decrease observed in the group receiving the highest dose of 500mg/kg (Figure 7). These findings suggest that the VAC extract may possess antioxidant properties and could potentially mitigate oxidative stress in the ovaries of POF mice. Further investigation is needed to elucidate the underlying mechanisms of this observed effect and its potential implications for ovarian health.

### FMR1 gene expression analysis

**Figure 8:**
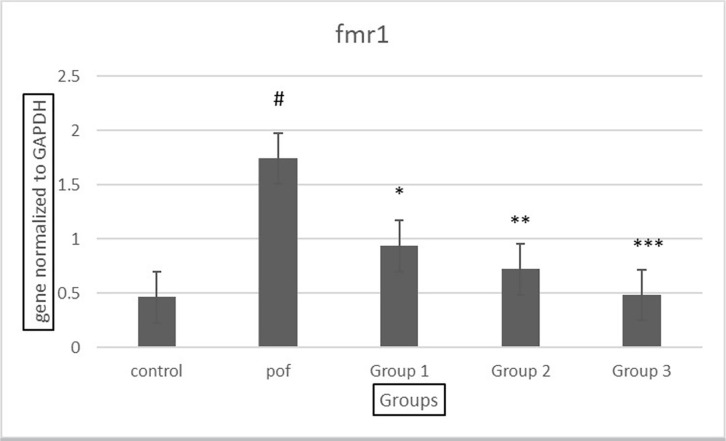
Comparison of fmr1 gene expression in all groups, group 1: POF+ 100 dose of *Vitex agnuscastus*, group 2: POF+ 300 dose of *Vitex agnus-castus*, group 3: POF+ 500 dose of *Vitex agnus-castus*.

The analysis of FMR1 gene expression revealed that in the POF group, there was a significant increase in the expression of the gene compared to the control group. However, with treatment using different doses of the VAC extract, the expression of the FMR1 gene decreased significantly compared to the POF group (*p*<0.05), with the most significant reduction observed at the highest dose of 500 mg/kg (*p*<0.001). Furthermore, no significant difference was observed in the expression of the FMR1 gene at the 500 mg/kg dose compared to the control group (Figure 8). These results suggest that the *Vitex agnuscastus* (VAC) extract may have a potential regulatory effect on the expression of the FMR1 gene, which is associated with premature ovarian failure. Further investigations are warranted to elucidate the molecular mechanisms underlying this observed effect and its potential therapeutic implications.

## DISCUSSION

In the present study, we investigated the impact of cyclophosphamide on the ovaries of NMRI mice, as well as the efficacy of *Vitex agnus-castus* plant extract treatment. After administering cyclophosphamide at a specific dose for 14 days, we observed that the mice began to lose weight at the same time that deaths happened in each of the groups. Furthermore, the ovary’s weight and oestrous cycle can be significantly impacted by cyclophosphamide treatment. Cyclophosphamide also damaged the ovarian tissue, disruption of the ovulation process, and induction of premature ovarian failure in mice.

Normal levels of reactive oxygen species (ROS) play an important role in regulating follicular growth, angiogenesis and sex hormone synthesis in ovarian tissue. When the balance between ROS and antioxidants is disrupted, however, it can cause serious consequences of oxidative stress (Shi *et al*., 2023). In line with earlier research, Injecting cyclophosphamide and developing POF disease in mice causes an increase in oxidative stress (Jia *et al*., 2022). Since cyclophosphamide is an alkylating agent that damages follicles and causes degeneration and fibrosis in ovarian tissue. It also induces oxidative stress (Inoue, 2022; Reckers *et al*., 2022). We observed an increase in FSH levels and a reduction in E2 levels, which supports the results of studies indicating similar patterns of hormonal changes in POF. (Bahrehbar *et al*., 2020)

The action mechanism of cyclophosphamide is that it is activated by liver enzymes and creates a covalent bond with the DNA of proteins and causes cell death. Cyclophosphamide specifically stimulates apoptosis in granulosa cells (Becker *et al*., 2005). Granulosa cells play a crucial role in the secretion of estrogen, and the apoptosis of these cells leads to a decrease in estrogen levels, since FSH receptors (FSHR) are mainly expressed in granulosa cells, apoptosis of granulosa cells causes the loss of the inhibitory effect on follicle-stimulating hormone (FSH) and its increase (Song *et al*., 2016). In agreement with previous studies, our findings similarly revealed that after administering cyclophosphamide and developing a model of premature ovarian failure, follicular counts were performed. The results indicated a significant decrease in the number of follicles across all categories following the drug administration (Erxian decoction alleviates cisplatin-induced premature ovarian failure in rats by reducing oxidation levels in ovarian granulosa cells).

Cyclophosphamide reduces the number of growing follicles in the ovarian tissue and increases the number of atretic follicles, leading to a decrease in the number of mature follicles and corpus luteum (Cox & Liu, 2014). In this study Similar to previous research, following the administration of cyclophosphamide to induce a premature ovarian failure model, irregularities in the estrous cycle were observed (Wang *et al*., 2020). Since the estrous cycle is controlled by estrogen and progesterone, in early ovarian failure, where we see a decrease in these two hormones, the normal process of the estrous cycle is disrupted, so that the proestrous phase is longer and the estrous phase is not observed. Female mice can only become pregnant when ovulation occurs (during the estrous phase) (Ojah *et al*., 2021). In brief, our results and previous studies showed that the expression level of FMR1 gene was investigated in premature ovarian failure disease and the results showed increased expression of FMR1 gene mRNA in ovaries (Halder *et al*., 2022). The FMR1 gene is critical for ovarian reserve and folliculogenesis (Rehnitz *et al*., 2021). It encodes the FMRP protein, this protein is mainly located in granulosa cells which is essential for creating synapses between neurons and regulating the production of other proteins. Importantly, FMRP is necessary for the proper functioning of the ovary. this protein is mainly located in granulosa cells (Sullivan *et al*., 2011; Tassone & Hagerman, 2003). Mutations in the FMR1 gene, located on the X chromosome, cause various diseases, including premature ovarian failure (Murray, 2000). Some studies suggest that changes in FMR1 transcript levels in premutation carriers in women contribute to the development of premature ovarian failure. The premutation leads to the overproduction of abnormal FMR1 mRNA that contains repeat expansions, which researchers believe causes the signs and symptoms of FXPOF. The mRNA is thought to bind to other proteins and prevent them from doing their functions (Oostra & Willemsen, 2003). The methanolic extract of the *Vitex agnus-castus* plant contains ligands for the estrogen receptor, resulting in increased estrogen levels after the gavage of the extract in the treatment groups (Chen *et al*., 2011). This plant also binds to the dopamine receptor in the pituitary and hypothalamus, inhibiting the secretion of prolactin. As an endocrine gland, the ovary’s secretion of progesterone increases with *Vitex agnus-castus* plant extract, inducing the natural formation of the corpus luteum (Dugoua *et al*., 2008). The plant’s diterpenes normalize the levels of estrogen, progesterone, and prolactin, while its antioxidant activity inhibits free radicals, reducing their amount in the treatment groups (Sağlam *et al*., 2007). Plants and their derivatives, such as flavonoids and alkaloids, have the potential to create biological immunity due to their bioactive molecules (Rashmeei *et al*., 2020). When the FMR1 gene is mutated, the FMR protein (FMRP) is expressed in a lower-than-normal amount and it is associated with an increase in transcription. The increase in mRNA production in the cells that express the gene additionally leads to the formation of inclusion bodies. Accumulation of inclusion bodies disturbs these cells’ normal function, leading to FXTAS in neurons and FXPOI in oocytes and granulosa cells. An increase in FMR1 mRNA and a decrease in FMRP level should be considered. Administering *Vitex agnus-castus* plant extract to mice decreased the mRNA level of the FMR1 gene, resulting in decreased gene expression (Abdi *et al*., 2021).

After creating a premature ovarian failure model using cyclophosphamide, we investigated the expression level of the FMR1 gene in all groups. Results showed a significant increase in FMR1 gene expression in the POF group compared to the healthy group. In the treatment groups with *Vitex agnus-castus* plant extract, the expression level of this gene decreased, with a more significant decrease observed in the 500 group, which was close to the control.

## CONCLUSION

According to this research, we induced a model of premature ovarian failure by using chemotherapy drug cyclophosphamide and we used the alcoholic extract of *Vitex agnus-castus* plant in three different doses. It has helped. Meanwhile, with the increase in the dose of the extract, a significant effect was observed in the healing process of this disease.

## ABBREVIATIONS

Premature ovarian failure: POF
*Vitex agnus-castus*: VAC
anti-Müllerian hormone: AMH
Fragile-X-Mental-Retardation-1: FMR1
Fragile X-associated tremor/ataxia syndrome: FXTAS
Animal Research Reporting of In Vivo Experiments: ARRIVE
Reactive Oxidative Stress: ROS
Follicle-stimulating hormone: FSH
Hematoxylin and eosin: H&E
Intraperitoneal: IP
